# Dr. James S. Trotter

**Published:** 1905-11

**Authors:** 


Dr. JAMES S. TROTTER, formerly of Buffalo, died at his home in Waterford, Ont., November 11, 1905. He was a graduate in medicine of the University of Buffalo, class of 1891, and immediately took up his residence and practice here until his health failed two or three years ago. He visited the Klondike region in the early part of the gold excitement there, but returned after a few months' absence. His funeral was held at Waterford November 14, and was attended by several Buffalo physicians.

